# Association between intracranial vessel calcifications, structural brain damage, and cognitive impairment after minor strokes: a prospective study

**DOI:** 10.3389/fneur.2023.1218077

**Published:** 2023-07-18

**Authors:** Estelle Emanuelle Seyman, Udi Sadeh-Gonik, Phillip Berman, Itay Blum, Genady Shendler, Bornstein Nathan, Ofer Rothschild, Jeremy Molad, Einor Ben Assayag, Hen Hallevi, the TABASCO prospective cohort study group

**Affiliations:** ^1^Stroke Department Division of Neurology, Tel Aviv Sourasky Medical Center, Tel Aviv, Israel; ^2^Sackler Faculty of Medicine, Tel Aviv University, Tel Aviv, Israel; ^3^Department of Radiology, Tel Aviv Sourasky Medical Center, Tel Aviv, Israel; ^4^Brain Center, Shaare Zedek Medical Center, Jerusalem, Israel; ^5^Sagol School of Neuroscience, Tel Aviv University, Tel Aviv, Israel

**Keywords:** ischemic stroke, cerebral small vessel disease, post stroke dementia, vascular calcifications, intracranial calcium score

## Abstract

**Background:**

Vascular calcifications are a hallmark of atherosclerosis, and in the coronary arteries are routinely used as a prognostic marker. Calcifications of intracranial vessels (ICC) are frequently observed on non-contrast CT (NCCT) and their effect on post-stroke cognitive impairment (PSCI) remains unclear. Our aim was to explore the association of ICC with prospective long-term cognitive function and advanced MRI-measures in a large prospective cohort of cognitively intact mild stroke survivors.

**Methods:**

Data from the Tel-Aviv brain acute stroke cohort (TABASCO) study [ClinicalTrials.gov #NCT01926691] were analyzed. This prospective cohort study (*n* = 575) aimed to identify predictors of PSCI, in cognitively intact mild stroke survivors. A quantitative assessment of the intracranial calcium content – The ICC score (ICCS) was calculated semi-automatically on NCCT using a validated calcium quantification application. Participants underwent a 3 T-MRI and prospective comprehensive cognitive clinical and laboratory assessments at enrollment, 6, 12, and 24-months.

**Results:**

Data were available for 531 participants (67.4 years, 59.5% males). The incidence of PSCI at two-years doubled in the high ICCS group (26% vs. 13.7%, *p* < 0.001). The high ICCS group had significantly greater small-vessel-disease (SVD) tissue changes and reduced microstructural-integrity assessed by Diffusion-Tensor-Imaging (DTI) maps (*p* < 0.05 for all). In multivariate analysis, a higher ICCS was independently associated with brain atrophy manifested by lower normalized white and gray matter, hippocampal and thalamic volumes (*β* = −0.178, *β* = −0.2, *β* = −0.137, *β* = −0.157; *p* < 0.05) and independently predicted PSCI (OR 1.83, 95%CI 1.01–3.35).

**Conclusion:**

Our findings suggest that the ICCS, which is a simple and readily available imaging marker on NCCT, is associated with brain atrophy, microstructural damage, the extent of SVD, and may predict PSCI. This finding has implications for identifying individuals at risk for PSCI and implementing targeted interventions to mitigate this risk.

## Background

Post-stroke cognitive impairment (PSCI) is a significant long-term complication affecting stroke survivors and contributing to the global burden of dementia (1). Risk factors for PSCI include age, lower education, socioeconomic disparities, pre-existing cognitive decline, and vascular risk-profile (2). Atherosclerosis, a common cause of vascular diseases (3) is strongly associated with cognitive dysfunction (4–8). Clinical assessment of atherosclerosis focuses on identifying risk factors for acute vascular events (9). These risk assessment tools fail to reflect the ongoing effects of atherosclerotic plaque formation and progression on adjacent brain tissue. The extent to which atherosclerosis exerts a chronic silent effect leading to tissue dysfunction is unclear.

Vascular calcifications are a surrogate for atherosclerosis and act as its established pathological feature in different vascular beds (10). Local inflammation, glycemic and lipid control promote osteogenic differentiation in vascular cells within the atheroma, causing mineralization (11). Recent publications showed correlation between the systemic atherosclerotic load, as assessed by quantitative calcium calculations, and cognitive function (12). The Coronary Calcium Score (CAC), e.g., Agatston score, is the best-studied vascular calcification and is a highly specific feature of coronary atherosclerosis (13–15). It correlates with cognitive decline, consistent with the hypothesis that vascular injuries play a role in the development of dementia (12, 16). Similarly, the extent of vascular calcifications in other arterial beds was shown to correlate with cognitive decline (12, 16, 17) and increased mortality (18). Intracranial calcifications (ICC) were first observed in the 1960s using *ex-vivo* radiography and pathology investigations. Although ICC are frequently observed on Non-Contrast CT(NCCT), their clinical significance was not investigated until recent years.

Neuroimaging features are important predictors for PSCI (19). Previous studies have primarily investigated magnetic resonance imaging (MRI) findings, such as the location and volume of stroke, measures of brain atrophy, and markers of cerebral small vessel disease (SVD) (19). Furthermore, intracranial stenosis, as detected by CT angiography (CTA), is also associated with an increased risk of PSCI (20, 21).

There is increasing evidence linking ICC to elevated stroke rates, imaging-verified SVD markers (22), and their influence on stroke outcomes following endovascular thrombectomy (23). However, there is currently a lack of data examining the association between intracranial calcifications as an imaging marker and the risk of future PSCI.

In this study, we employed a semi-automated quantitative calculation of the ICC to create the ICC-score (ICCS), allowing us to quantify the calcium load in the main intracranial vessels in a large prospective cohort of cognitively intact mild stroke survivors.

Our objective was to investigate the potential association between ICCS and a range of radiological and clinical outcomes, including brain atrophy, microstructural integrity, SVD markers and long-term cognitive function, and asses its utility as an imaging marker for PSCI.

## Methods

This study represents an analysis of data obtained from a single-center prospective cohort study known as the Tel-Aviv-brain-acute-stroke cohort (TABASCO) (ClinicalTrials.gov #NCT01926691), whose detailed design and protocol have been previously described (24). Patients included were men and women over 50 years old, admitted within 72 h after a first-ever acute ischemic stroke or transient ischemic attack (TIA), with a total NIH Stroke Scale (NIHSS) <17. Exclusion criteria: stroke that resulted from trauma/invasive procedures, hemorrhagic stroke, a diagnosis of dementia or cognitive impairment before the stroke (determined by Informant Questionnaire on Cognitive Decline in the Elderly score > 3.3) (25), severe aphasia or disability which made the possibility of follow-up unlikely.

**Figure 1:**
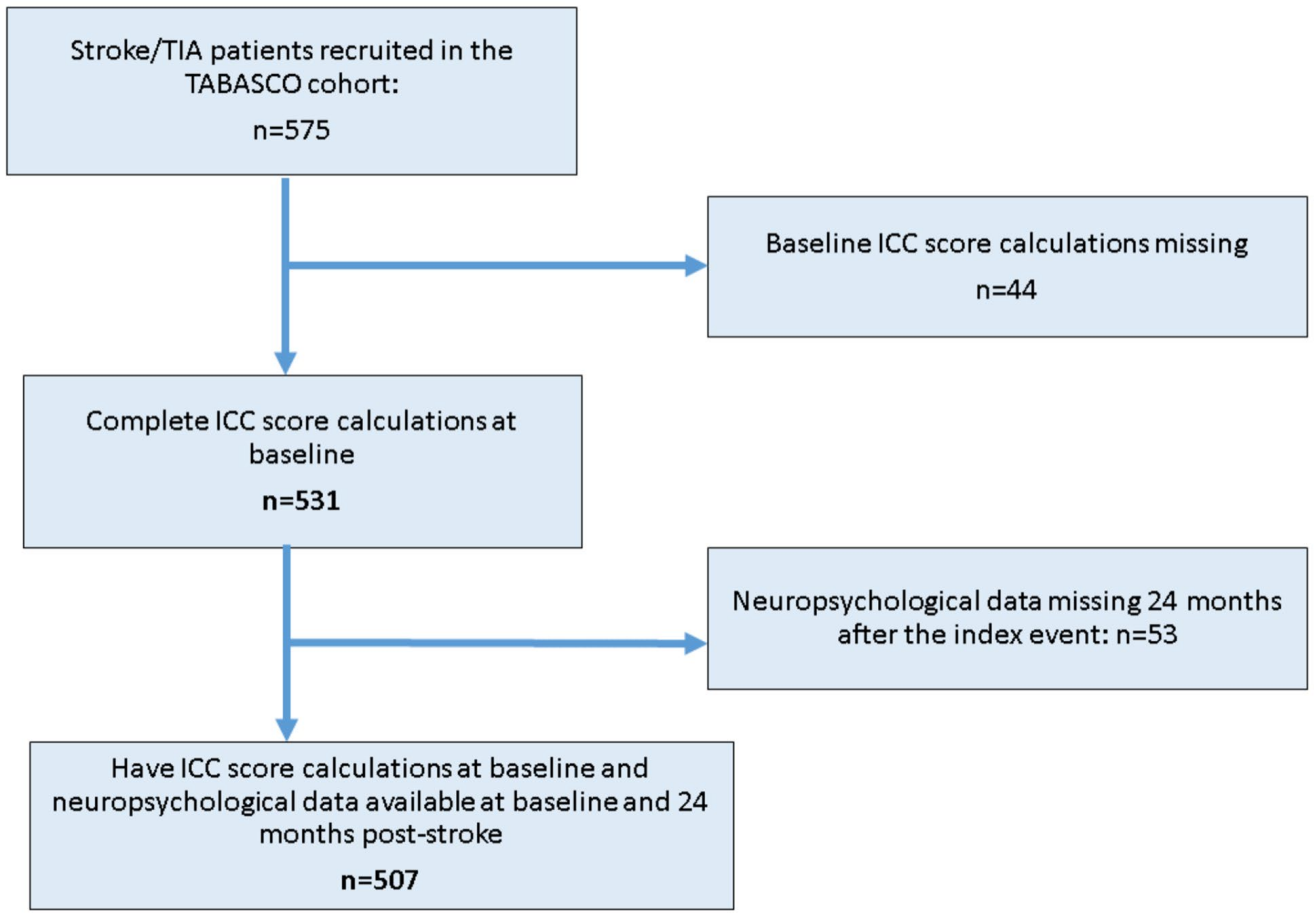
Study flow chart of patient selection for the current analysis ICC-Intra cranial calcium, TIA, transient ischemic attack; TABASCO, Tel Aviv brain acute stroke cohort.

The cohort consisted of 575 consecutive eligible patients who were recruited between April 1st, 2008 and December 1st, 2014, with a prospective follow-up period ranging from 2 to 8 years. Figure 1 shows the flow chart of patient and data selection for this current analysis.

All participants signed informed consent forms, approved by the local ethics committee.

Patients completed a baseline neuropsychological assessment including the Montreal Cognitive Assessment(MoCA) (26) and the NeuroTrax computerized cognitive testing (NeuroTrax Corp., Bellaire, TX) (27) during the first days after the stroke. These comprehensive neuropsychological evaluations were repeated at 6, 12, and 24 months (28). A Global Cognitive Score was computed as the average of the six index scores (memory, executive functions, visuospatial perception, verbal function, attention and motor skills) as previously described (28). All scores were normalized according to stratifications of age and education to give a distribution with a mean of 100 and a standard deviation of 15. All cognitive assessments were done using different validated versions for repeated measures. Complete cognitive assessments 2-years after the index event were available for 507 (88%) participants due to loss of follow-up. Additionally, detailed neuro-status and laboratory assessments were performed throughout the follow-up period, including polymorphism of genes of interest such as Apolipoprotein E4 (ApoE) as previously describe (29).

### Criteria for cognitive impairment

Participants who demonstrated signs of cognitive impairment based on the cognitive tests mentioned above were referred to a highly experienced cognitive neurologist, along with their caregiver, for a comprehensive evaluation. These assessments were further reviewed by a consensus forum to determine whether the participant had dementia, mild cognitive impairment (MCI), or was cognitively intact (29). The forum consisted of the assessor, three senior neurologists specializing in memory disorders with 7–10 years of experience, and a neuropsychologist with 10 years of experience. Post-stroke cognitive impairment (PSCI) was defined as a diagnosis of either MCI or dementia throughout the 24-month follow-up period.

### NCCT image acquisition and analysis

All study participants underwent NCCT on hospital admission. NCCT acquisition was performed according to standard departmental protocols with 8- or 16-section multidetector CT scanners (GE Healthcare), slice thickness 0.3 mm, with the patient in a head holder in transverse plane.

### ICC score analysis

NCCT scans were transferred to a PC-based Philips portal workstation for further analysis. The calcium quantification analysis was performed using the Philips portal software HeartBeat CS application version 9 in a semi-automated manner. The readers were blinded to all clinical and radiological data, including stroke location and volume. To ensure the accuracy of the results, inter-observer reliability was assessed by evaluating 15 scans, yielding an intra-class correlation score of 0.98 (95% CI 0.961–0.996).

**Figure 2:**
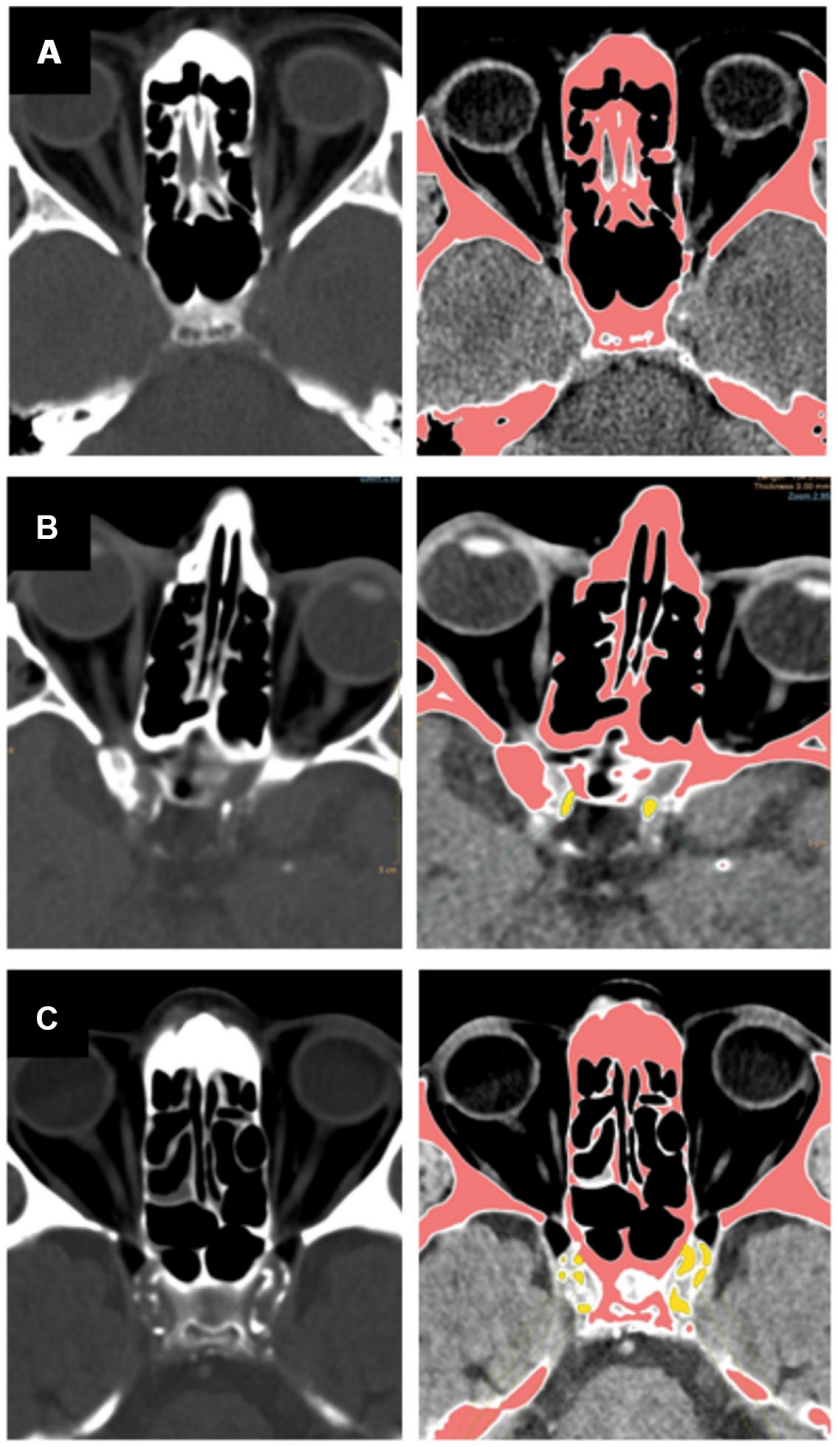
Presented are demonstrative images showcasing quantitative analysis of the intracranial calcium score, depicting axial non-contrast CT scan images, bone window, and the semi-automated quantitative ICC analysis. Hyper-attenuated regions indicating calcifications are depicted in pink, vessel calcifications are outlined manually in yellow. Panel **(A)** – absence of calcifications with a low ICCS, Panel **(B)** – moderate burden of intracranial calcifications with a moderately increased ICCS, Panel **(C)** – high burden of intracranial calcifications resulting in a high ICCS.

Experienced vascular neurologist (ES) and neuro-radiologists (US, SG, IB) calculated the ICCS by analyzing NCCT images in axial planes with a bone window. The ICCS was obtained by summing the calcium content in the proximal intracranial arteries, which include the internal carotid, vertebral, and basilar arteries. The Agatston method (14, 15) was used to analyze areas with a density greater than 130 Hounsfield units (Hu). The software automatically highlighted areas with a density greater than 130 HU, as shown in Figure 2. A region-of-interest (ROI) was manually contoured in each of the aforementioned vessels in all axial slices showing the vessel to measure the peak attenuation of all intracranial vessels. The readers adjusted the window/level and followed the anatomical contour of the vessels to ensure only vessels were included in the analysis. All ROIs were carefully examined on zoomed images and manually contoured to ensure that the bony base-of-skull was not incorporated in the analysis.

The ICCS was obtained for each artery by multiplying the plaque area by a weighted peak attenuation score, as routinely done when calculating the Agatston score. The weighted peak attenuation was calculated using a cofactor of 1–4, depending on the Hounsfield unit range: cofactor 1 (130–199 Hu), cofactor 2 (200–299 Hu), cofactor 3 (300–399 Hu), and cofactor 4 (>400 Hu). The total ICCS was obtained by summing the scores for each vessel.

### CTA image acquisition and analysis

A subgroup of study participants (*n* = 194) underwent a cranio-cervical CTA, as part of routine clinical workup, done at the discretion of the treating physician. All CTA exams were obtained with a Philips helical scan technique on the same scanner. Coverage was from the aortic arch to vertex, with straight axial sections parallel to the orbitomeatal line with 0.9 -mm section thickness, 120 kV. Acquisitions were obtained after a single bolus intravenous contrast injection of 80 mL contrast media into an antecubital vein at 3–5 mL/s, auto-triggered by appearance of contrast in a ROI placed in the ascending aorta. Experienced stroke neurologists (ES, HH) and neuroradiologists (US, IB, GS) assessed the degree of luminal stenosis in the cranio-cervical vessels on bone window axial images. All vessels were viewed at predetermined locations, which were verified on coronal and sagittal planes. The vessels were visually assessed and graded if they had significant stenosis (>50%) in any of the predetermined segments. The sum of stenotic segments in each vessel and location constituted the overall assessment of cranio-cervical vessel stenosis.

### MRI image acquisition and analyses

All images were acquired within seven days of stroke onset on a 3T GE scanner (GE Signa EXCITE, Milwaukee, WI, USA) using an 8-channel head coil. The acquisition protocol included the following pulse sequences: Axial fast Spin-Echo (FSE) T2-weighted images (WI) [Time to repeat (TR)/Time to echo (TE) = 13,000/110milliseconds (msec.)], Field of View (FOV) = 240, Matrix = 512×256 and slice thickness of 4 mm with no gap, fluid-attenuated inversion recovery (FLAIR), T2* Gradient Echo (GE) image, and high-resolution 3D T1-WI spoiled GE sequences (SPGR) axial (TR/TE = 8.976/3.488 msec, FOV = 256, Matrix = 256×256 and slice thickness of 1 mm) and coronal (TR/TE = 6.35/1.8 msec, FOV = 250, Matrix = 256×256 and slice thickness of 2 mm). Diffusion-weighted imaging (DWI) sequence (TR/TE = 6000/72.4 msec, FOV = 240, Matrix = 128×128 and slice thickness of 4 mm with no gap, b values = 0, 1,000 s/mm) and diffusion tensor images (DTI) were obtained using echo planar imaging (EPI) sequence. All axial slices were prescribed on the same orientation, covering the whole brain, aligned along the fourth ventricle-orbitofrontal orientation.

#### Ischemic infarct identification and volume

A senior neuroradiologist (US) assessed the presence of acute ischemic infarcts based on the DWI images. Cortical infarcts were defined as any infarct involving the cortex. Sub-tentorial infarcts were defined as cerebellar or brainstem infarctions. The volumes (mm3) of the ischemic lesions were calculated. The ischemic lesions were quantified using a semi-automatic method (30).

#### Small vessel disease markers

All MRI scans were thoroughly evaluated for SVD radiological markers according to the STRIVE protocol (31) including (A) White matter hyperintensities (WMH) were graded using the Fazekas score system (32, 33). (B) Lacunes were defined as sharply demarcated hypo-intense lesions sized between 3 mm and 15 mm in diameter on T1-weighted images with corresponding hypo intense lesions with hyper-intense rim on T2 dark fluid (31). (C) Cerebral microbleeds (CMB) were defined as round hypo intense lesions on SWI with a diameter < 10 mm. Other sequences including DWI and T1 were evaluated to rule out CMB mimics. CMBs were divided into lobar versus deep (31, 34). (D) Enlarged perivascular spaces (PVS) were defined as smooth margin, round, oval, or linear-shaped lesions, sized up to 3 mm, with signal intensity equal to cerebrospinal fluid on T2-weighted images. Enlarged perivascular spaces at the basal ganglia level and the centrum semiovale in the most involved hemisphere were counted (31, 35). We counted enlarged perivascular spaces in the most affected hemisphere. One point was awarded if 30 or more enlarged perivascular spaces were present.

#### Tissue segmentation

The identification and quantification of the ischemic lesions, total gray matter (GM) volume, WMH, and normal-appearing white matter (NAWM) were performed using a multi-modal view with a semi-automatic method (30). Volumes (mm3) of ischemic lesions and WMH were calculated across the whole brain. All volumes were normalized by dividing the total volume of each tissue cluster by the intracranial volume (ICV).

#### Detailed volumetric measurement

The volumetric analysis was performed on high-resolution 3D T1-WI axial images. Analysis was performed using the FreeSurfer V5.1 image analysis suite^1^ for all brain segmentation based on probabilistic atlas and intensity values. The automated procedure includes skull-stripping, intensity normalization, Talairach transformation, tissue segmentation, and surface tessellation (36, 37). The complete FreeSurfer analysis pipeline was performed with manual intervention and data quality assurance. Based on the automated segmentation, we extracted each subject’s left and right hippocampi and thalamus volumes, normalized to ICV.

#### Diffusion-tensor-imaging analysis of the NAWM

Diffusion-tensor-imaging (DTI) maps calculation was performed using FMRIB Diffusion Toolbox, part of FMRIB Software Library (FSL),^2^ and included eddy current and motion correction. Four different maps: fractional anisotropy (FA), mean diffusivity (MD), axial and radial diffusivities (λ⊥, λ||) were calculated.

#### Hippocampal MD

Hippocampal DTI analyses were performed using FreeSurfer software.^3^ Motion and eddy current corrections were made before calculating DTI indices. All images were registered to the T2-weighted low b value (b50) image. A rigid transform was computed that maximizes the mutual information between the T1-weighted anatomical and the T2-weighted low-b image (38). The low b value image was registered to each subject’s anatomical volume, and the MD map was analyzed in register with the low-b. Left and right hippocampi VOIs were individually masked with the FreeSurfer results. Hippocampi masks were eroded to avoid partial volume effects near the GM/WM border or CSF. For each patient, for each VOI, MD values were averaged across all voxels.

### Statistical analysis

Analyses were performed using SPSS version 27.0. (SPSS Inc. Chicago, IL). Student’s *t*-test were used to assess significant differences in clinical and radiological data in patients with high (above median) versus low (below median) ICCS. Categorical data was analyzed on Fisher exact test. Missing data were filled in with a multiple imputation method under the “missing at random” assumption. For regression analyses, both MCI and dementia patients were grouped together as a PSCI. Multivariate linear regressions models were used to explore associations between ICCS and results of cognitive, functional and MRI outcome measures, while controlling for possible confounders: age, gender, education years and vascular risk factors: history of hypertension, T2DM, dyslipidemia. Multi-collinearity was checked by variance inflation factor (VIF), with VIF <5 and tolerance >0.1 considered to indicate no significant collinearity, and the potentially most performing parameters were identified. Further, to determine univariate proportional hazard ratios for each risk factor, univariate logistic regression models were employed from index stroke to development of cognitive impairment 24 months post-stroke (PSCI risk) as the dependent variable. All variables with a value of *p* <0.25 were taken into the multivariable model to minimize the possible confounders. The multi-collinearity test was carried out to see the correlation between independent variables by using a VIF (as dyslipidemia correlated with ICCS, we only included ICCS in the model). Of note: no significant collinearity observed between dyslipidemia and brain atrophy, microstructural tissue damage, brain small vessel disease and PSCI, (although they are clinically interrelated). The odds ratio along with 95% CI were estimated to measure the strength of association and to identify factors associated with PSCI risk using multivariable logistic regression. Based on multivariable logistic regression all variables with a value of *p* less than 0.05 on bivariate results were taken as a significant determinant of PSCI risk.

## Results

A total of 575 consecutive, cognitively intact patients admitted through the emergency medicine department at the Tel-Aviv Medical Center between April 1st 2008 and December 1st 2014, within 72-h of symptom onset, were recruited and comprised the TABASCO cohort. ICCS calculations were not available for 44 (8%) participants, due to missing or technically inadequate NCCT. These participants did not differ from the whole cohort with regards to baseline characteristics. The mean age of the entire cohort was 67.4 years, 59.5% male, with a median ICCS of 57.1 (IQR 3.46–248.13). The ICCS was greater in the anterior circulation versus the posterior circulation vessels (mean ICCS 180.8 versus 8.02 for anterior versus posterior circulation vessels respectively, *p* < 0.001).

### Baseline demographic and clinical characteristics

Table 1 presents a comprehensive overview of the demographic and clinical characteristics of subjects categorized into high (above median) and low (below median) ICCS groups. Participants with higher ICCS were older (mean age 71.5 ± 9.4 vs. 63.6 ± 8.9 years, *p* < 0.001), less educated (mean education years of 12.7 ± 3.8 vs. 13.5 ± 3.7, *p* = 0.016) had higher BMI (mean BMI of 27.8 ± 5.1 vs. 26.8 ± 4.1, *p* = 0.031), higher systolic blood pressure (BP) (mean systolic BP 151.3 ± 24 vs.146 ± 23.9, *p* = 0.011) and overall higher Framingham risk scores (mean score 13.9 ± 5.0 vs. 9.5 ± 5.4, p < 0.001) compared to the low ICCS group, respectively. Furthermore, the higher ICCS group displayed more severe stroke symptoms at admission (median NIHSS of 2[1–5] vs. 2[0–4], *p* = 0.001), with higher rates of large artery atherosclerosis (10.4% vs. 3.9%, *p* = 0.029) compared to the lower ICCS group, respectively.

**Table 1:** Baseline and follow up characteristics of study cohort (*n* = 531).

	Low ICC score	High ICC score	*p* value
Clinical characteristics	*N* = 265	*N* = 266	
Age, years (SD)	63.6 (8.9)	71.5 (9.4)	**<0.001**
Female gender, *n* (%)	152 (57.4)	164 (61.7)	0.345
Education, years (SD)	13.5 (3.7)	12.7 (3.8)	**0.016**
Body-mass index, kg/m2 (SD)	26.8 (4.1)	27.8 (5.1)	**0.031**
Systolic blood pressure, mmHg (SD)	146 (23.9)	151.3 (24)	**0.011**
Ever smoked, *n* (%)	164 (61.9%)	170 (63.9%)	0.661
T2DM, *n* (%)	62 (23.4)	97 (36.7)	**0.001**
Dyslipidemia, *n* (%)	133 (50.2)	155 (58.7)	**0.049**
Hypertension, *n* (%)	138 (52.1)	188 (71.2)	**<0.001**
Framingham Risk Score for Stroke	9.5 (5.4)	13.9 (5.0)	**<0.001**
Admission NIHSS, median (IQR)	2 (0–4)	2 (1–5)	**0.001**
TIA (%)	84 (31.7)	65 (24.4)	0.065
Stroke Etiology (TOAST criteria)	*N* = 181	*N* = 201	
Lacunar strokes; *n* (%)	89 (49.2%)	96 (47.8%)	0.795
Cardioembolic stroke; *n* (%)	26 (14.4%)	21 (10.4%)	0.259
Large-artery atherosclerotic stroke; *n* (%)	7 (3.9%)	21 (10.4%)	**0.029**
Other or undetermined etiology; *n* (%)	27 (14.9%)	33 (16.4%)	0.764
Brain CT parameters			
Total ICC, median (IQR)	3 (0–18.6)	240 (122–502)	**<0.001**
Global stenosis assessment^*^, mean(SD)	0.50 (0.86)	0.88 (1.21)	0.07
Brain MRI parameters	*N* = 210	*N* = 189	
No infarct in MRI, *n* (%)	78 (37.1%)	41 (21.7%)	**0.002**
Cortical infarct, *n* (%)	53 (25.2%)	49 (25.9%)	0.946
Sub-cortical infarct, *n* (%)	53 (25.2%)	75 (39.7%)	**0.004**
Sub-tentorial infarct, *n* (%)	26 (12.4%)	24 (12.7%)	0.964
White matter hyperintensity (Fazekas) score, median (IQR)	0 (0–1)	1 (0–1)	**<0.001**
Ischemic lesions volume, mm^3^; mean (SD)	3309.7 (9416.8)	4197.4 (9985.8)	0.495
Total WM volume, normalized to ICV; mean (SD)	31.4 (3.0)	29.5 (2.9)	**<0.001**
Total GM volume, normalized to ICV; mean (SD)	40.8 (3.3)	38.7 (3.1)	**<0.001**
Total thalamus volume, normalized to ICV; mean (SD)	0.84 (0.1)	0.79 (0.08)	**<0.001**
Total hippocampal volume, normalized to ICV; mean (SD)	0.54 (0.08)	0.50 (0.08)	**<0.001**
Hippocampi mean diffusivity, mm^2^/s; mean (SD)	0.0012 (0.00012)	0.0013 (0.00011)	**<0.001**
Frontal cortex thickness, mm^2^; mean (SD)	2.43 (0.13)	2.38 (0.13)	**<0.001**
Temporal cortex thickness, mm^2^; mean (SD)	2.73 (0.17)	2.62 (0.17)	**<0.001**
Parietal cortex thickness, mm^2^; mean (SD)	2.28 (0.12)	2.20 (0.13)	**<0.001**
Occipital cortex thickness, mm^2^; mean (SD)	2.01 (0.13)	1.95 (0.12)	**<0.001**
NAWM MD values, mm^2^/s; mean (SD)	0.0008 (0.00004)	0.0009 (0.00006)	0.101
NAWM FA values, arbitrary units; mean (SD)	0.4046 (0.1222)	0.40 (0.1302)	**<0.001**
NAWM λ|| values, mm^2^/s; mean (SD)	0.00066 (0.00004)	0.00068 (0.00005)	**<0.001**
NAWM λ⊥ values, mm^2^/s; mean (SD)	0.00123 (0.00005)	0.00126 (0.00007)	**<0.001**
Lobar Microbleed; *n* (%)	16 (6.0%)	33 (12.4%)	**0.011**
Deep Lobar Microbleed; *n* (%)	5 (1.9%)	13 (4.9%)	0.056
Lacunes; *n* (%)	65 (31%)	87 (46%)	**0.002**
Perivascular spaces; *n* (%)	198 (94.3%)	164 (86.8%)	**0.014**
Prospective Cognitive scores			
Admission MoCA score; mean (SD)	24.1 (3.3)	22.7 (3.8)	**<0.001**
MoCA score at 6 months; mean (SD)	25.6 (3.2)	24.3 (3.8)	**<0.001**
MoCA score at 12 months; mean (SD)	25.7 (3.2)	24.1 (4.2)	**<0.001**
MoCA score at 24 months; mean (SD)	25.6 (4.0)	23.6 (4.7)	**<0.001**
Computerized Total cognitive score at admission; mean (SD)	93.8 (13.2)	89.0 (14.0)	**0.002**
Computerized Total cognitive score 6 months post-stroke; mean (SD)	96.5 (12.4)	91.8 (12.2)	**<0.001**
Computerized Total cognitive score 12 months post-stroke; mean (SD)	97.6 (12.6)	93.6 (12.3)	**0.004**
Computerized Total cognitive score 24 months post-stroke; mean (SD) (*n* = 507)	98.3 (11.1)	93.9 (12.2)	**0.001**

Detailed demographic, clinical characteristics, neuroimaging data and cognitive outcomes in the high (above median) versus low (below median) ICC score groups. Entries are mean (SD) or *n* and %, as indicated. Significant results are shown in bold (*p* < 0.05). ^*^Global assessment of stenosis in the cranio-cervical vessels on CTA. λ⊥, axial diffusivity; λ||, radial diffusivity; FA, fractional anisotropy; GM, gray matter; ICV, intracranial volume; IQR, interquartile range; MD, mean diffusivity; MoCA, Montreal Cognitive Assessment; MRI, Magnetic resonance imaging; NAWM, normal-appearing white matter; NIHSS, National Institutes of Health Stroke Scale; SD, standard deviation; T2DM, Type 2 diabetes mellitus; TIA, transient ischemic attack; WM, white matter.

### ICCS and detailed MRI measures

MRI-neuroimaging data was absent for 132 (22%) of study cohort due to participant refusal, claustrophobia and technically inadequate acquisition. Notably, however, the incidence of PSCI was similar among those who did and did not have brain MRI scans at baseline (19.2% versus 19.7% respectively). Table 1 presents univariate analysis results of detailed, advanced MRI analyses in the low versus high ICCS groups. On univariate analysis, the high ICCS group demonstrated significant brain atrophy (mean normalized WM volume 29.5 ± 2.9 vs. 31 ± 3.0, *p* < 0.001; mean normalized GM volume 38.7 ± 3.1 vs. 40.8 ± 3.3, p < 0.001). Detailed volumetric analysis showed reduced frontal, temporal, parietal and occipital cortical thickness in the higher versus lower ICCS groups (*p* < 0.001 for all). Additionally, microstructural damage, assessed by DTI analysis (FA, λ⊥, and λ|| maps) of the NAWM, as well as all SVD markers were significantly higher in the high ICCS group (complete data presented in Table 1).

On multivariable regression models, where advanced MRI measures were included as the dependent variable and the ICCS was included as the independent variable of interest, while adjusting for potentially confounding variables including: age, gender, education and vascular risk factors: higher ICCS was independently associated with brain atrophy as manifested by lower normalized WM, GM, hippocampal and thalamic volumes (*β* = −0.178, *β* = −0.2, *β* = −0.137, *β* = −0.157; *p* < 0.05 for all) and with increased microstructural damage as assessed by NAWM λ|| maps (*β* = 0.13, *p* = 0.05). Multiple regression model data are presented in Table 2.

**Table 2:** Multiple regression models exploring the relation between ICC score, advanced MRI measures, and long-term cognitive function.

ICC score and MRI measures	Model 1			Model 2		
	*β*	SE	*p*	*β*	SE	*p*
Total WM volume, normalized to ICV	−0.31	0.32	**<0.001**	−0.18	0.34	**0.001**
Total GM volume, normalized to ICV	−0.32	0.35	**<0.001**	−0.20	0.36	**<0.001**
Total hippocampus volume, normalized to ICV	−0.24	0.01	**<0.001**	−0.14	0.01	**0.01**
Total thalamic volume, normalized to ICV	−0.25	0.01	**<0.001**	−0.16	0.01	**0.006**
Frontal cortex thickness	−0.20	0.01	**<0.001**	−0.09	0.02	0.15
Temporal cortex thickness	−0.31	0.02	**<0.001**	−0.16	0.02	**0.004**
Parietal cortex thickness	−0.28	0.01	**<0.001**	−0.15	0.01	**0.008**
Occipital cortex thickness	−0.21	0.01	**<0.001**	−0.11	0.01	0.07
Total NAWM volume	−0.16	11504.4	**0.014**	−0.07	10259.3	0.26
Ischemic lesion volume, mm^2^	0.05	1299.5	0.49	__	__	__
NAWM FA values, mm^2^/s	−0.11	0.002	0.10	__	__	__
NAWM MD values, mm^2^/s	0.26	<0.001	**<0.001**	0.12	<0.001	0.06
NAWM λ|| values, mm^2^/s	0.27	<0.001	**<0.001**	0.13	<0.001	**0.05**
NAWM λ⊥ values, mm^2^/s	0.24	<0.001	**<0.001**	0.12	<0.001	0.07

ICC score and cognitive function	Model 1			Model 3		
	*β*	SE	*p*	*β*	SE	*p*
Total cognitive score at admission	−0.17	1.51	**0.002**	−0.14	1.71	**0.02**
Total cognitive score at 6 months	−0.19	1.32	**<0.001**	−0.12	1.45	**0.03**
Total cognitive score at 12 months	−0.16	1.36	**0.004**	−0.12	1.41	**0.04**
Total cognitive score at 24 months (*n* = 507)	−0.19	1.32	**0.001**	−0.1	1.42	0.15

Multiple regression models exploring the association between the ICC score, advanced brain MRI measures and long-term cognitive function. Model 1 – unadjusted. Model 2 – adjusted for age, gender, education years and vascular risk factors: history of hypertension, T2DM, dyslipidemia. Model 3 – adjusted for age, gender, education years, stroke severity (NIHSS) and the presence of acute lesion in MRI. Significant results (*p* < 0.05) are shown in bold. ICC, Intra cranial calcium; MRI, magnetic resonance imaging; DA, axial diffusivity; λ⊥, axial diffusivity; λ||, radial diffusivity; GM, gray matter; ICV, intracranial volume; MD, mean diffusivity; NAWM, normal-appearing white matter; SE, standard error; T2DM, Type 2 diabetes mellitus; WM, white matter; TIA, transient ischemic attack; NIHSS, National Institutes of Health Stroke Scale. Entries are mean (SD) or *n* and %, as indicated. Significant results are shown in bold (*p* < 0.05).

### ICC score and longitudinal cognitive follow up

**Figure 3:**
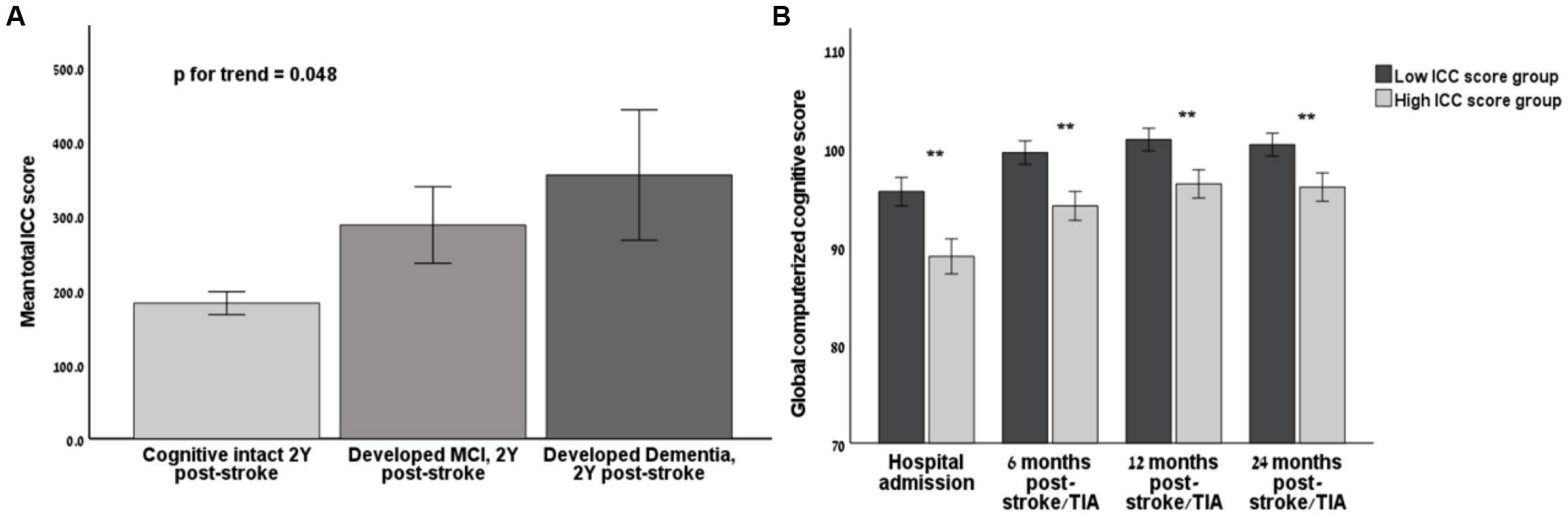
The association of Intra-Cranial-Calcium score and long-term cognitive outcomes **(A)** Bar chart demonstrating the mean Intra-Cranial-Calcium (ICC) score according to cognitive status at the end of longitudinal follow-up (2 years after the index event). **(B)** Bar chart demonstrating longitudinal cognitive function, as assessed by the global cognitive score of the computerized NeuroTrax battery, stratified according to high (above median) and low (below median) ICC score on NCCT at baseline on each time point post index event. ICC, Intra cranial calcium; Y, years; NCCT, non-contrast computed tomography; TIA, Transient ischemic attack; MCI, minimal cognitive impairment; SD, standard deviation. Line marks indicate SD limits. Significant results (*p* < 0.05) are marked by ^*^.

During the two years follow-up period, 105 participants (19.8%) developed clinically significant PSCI, as defined in the Methods. Of these, 16 patients (3.0%) developed dementia, and 89 patients (16.8%) developed MCI. The incidence of PSCI at the two-year time mark was doubled in the high ICCS group (26% vs. 13.7%, *p* < 0.001). The group who later developed MCI had significantly higher ICCS than the group who remained cognitively intact during the follow-up period of two years post-stroke (287.2 ± 471.2 vs. 182.1 ± 305.1, respectively, *p* = 0.03), while the group who later developed dementia had even higher ICCS at baseline (354.3 ± 350.6, *p* for trend = 0.048, Figure 3). Moreover, the ICCS correlated with worse cognitive function at all time points (*r* = −0.215, *r* = −0.245, *r* = −0.205, *r* = −0.221, respectively, *p* < 0.001for all).

On multivariable regression models, where cognitive scores were included as the dependent variable and the ICCS was included as the independent variable of interest, while adjusting for potentially confounding variables including: age, gender, vascular risk factors, NIHSS and the presence of an acute lesion on MRI: a higher ICCS was independently associated with lower cognitive outcomes at baseline, 6- and 12-month time marks (*β*= −0.14, *β* = −0.12, *β* = −0.12, respectively; *p* < 0.05 for all).The detailed regression model exploring the association between PSCI and ICCS is presented in Table 2.

Thirty seven percent of study cohort (*n* = 194) had a cranio-cervical CTA done as part of routine clinical care. Participants who had a CTA were younger (66.3 ± 9.5 vs. 68.1 ± 10.1, *p* = 0.035), had lower BMI (mean BMI of 26.3 ± 4.2 vs. 27.8 ± 4.8, *p* < 0.001) and had higher NIHSS (median NIHSS of 3[1–5] vs. 2[0–3], *p* < 0.001) compared to those who did not undergo CTA. No difference observed in the prevalence of vascular risk factors. An exploratory analysis of this group revealed that the ICCS correlated with the global stenosis assessment of the cranio-cervical vessels (*r* = 0.95, *p* < 0.001). Importantly, in participants with no evidence of vessel stenosis visible on CTA, the ICCS still negatively correlated with the global cognitive score at the end of the follow-up period (24-months post stroke) (*r* = −0.510, *p* = 0.003).

### Univariate and multivariate predictors of PSCI

Univariate and multivariate predictors for cognitive impairment in two years follow-up period are shown in Table 3. Univariate predictors included age (≥ 75), white matter hyperintensity (Fazekas) score (39), history of dyslipidemia, stroke severity and high ICCS. Since collinearity among dyslipidemia and ICCS was detected, dyslipidemia was excluded from the regression model. In multivariate analysis predicting cognitive impairment, predictors retained were age ≥ 75, stroke severity and high ICCS (OR 1.83, 95% CI 1.01–3.35) (Table 3).

**Table 3:** Univariate and multivariate predictors of post-stroke-cognitive-impairment (PSCI) within 24 months from stroke.

Baseline characteristic	Cognitive decline relative hazard ratio (95% CI)
Univariate predictors	
Age ≥ 75 years	**3.51 (2.21–5.57)**
Male gender	1.40 (0.90–2.18)
New lesion in MRI at hospital admission	1.89 (0.94–3.83)
Infarct volume	1.01 (0.98–1.04)
Stroke severity	**2.84 (1.58–5.11)**
White matter hyperintensity (Fazekas) score	**1.65 (0.99–2.74)**
History of dyslipidemia	**1.74 (1.10–2.75)**
History of hypertension	1.57 (0.99–2.51)
Ever smoked	1.02 (0.63–1.63)
APOE ε4 allele	1.12 (0.62–2.01)
High ICC score at hospital admission	**2.22 (1.39–3.54)**

Multivariate predictors	Relative hazard ratio (95% CI)	*p*
Age ≥ 75 years	**2.90 (1.58–5.32)**	**<0.001**
White matter hyperintensity (Fazekas) score	1.01 (0.74–1.37)	0.956
Stroke severity	**1.83 (1.01–3.35)**	**0.016**
High ICC score at hospital admission	**1.83 (1.01–3.35)**	**0.048**

Univariate and multivariate predictors of post-stroke-cognitive-impairment (PSCI) within 24 months from stroke. PSCI was defined as a diagnosis of either MCI or dementia throughout the 24 months follow-up. CI, confidence interval; ICC, Intra cranial calcium; MRI, Magnetic Resonance Imaging. Significant results are shown in bold (*p* < 0.05).

## Discussion

In this study, we assessed the extent of intracranial calcifications using the ICCS in a large prospective cohort of cognitively intact mild stroke survivors. We investigated the relationship between ICCS and advanced brain MRI analyses, as well as long-term cognitive measures. In our cohort, ICCS was associated with MRI-proven tissue damage at the time of enrollment including: cerebral SVD markers, brain atrophy, and NAWM microstructural damage. Additionally, ICCS was significantly associated with worse cognitive function at all time points up to 24-months post stroke, and higher scores were linked with a higher likelihood of developing PSCI. This score independently predicted PSCI 2-years after the index event.

PSCI is a common, devastating, and poorly understood complication of stroke, affecting up to 30% of stroke survivors (11). While it is traditionally related to age, stroke severity, and vascular risk factors (2), it is only partially explained by additional infarcts following the index event or secondary degeneration of brain structures. In our study population of minor stroke survivors who were cognitively intact at the time of image acquisition, ICCS showed a significant association with all SVD markers, brain atrophy, and microstructural damage, independent of age, gender, and vascular risk factors. These findings align with previously published data showing a correlation between ICCS and sub-cortical tissue damage, specifically SVD markers (40). Our study also found that ICCS independently correlated with microstructural damage in the NAWM and brain atrophy, specifically in brain structures crucial for cognitive function, such as the thalamus and hippocampus. Notably, there are no published studies assessing the ICCS in association with such detailed brain atrophy measures and DTI analyses.

The ICCS is a reliable measure of intracranial arterial calcifications on NCCT imaging and is considered a hallmark of atherosclerosis (10). Consistent with previous literature on vascular calcification in intracranial and other vascular beds (13, 17), we found that individuals with high ICCS also had a higher vascular risk factor profile, including diabetes and hypertension, a greater likelihood of large vessel atherosclerosis and more severe strokes (41). However, even after adjusting for cardiovascular risk profile, individuals with a higher ICCS still demonstrated an association with greater MRI proven tissue damage, significant detailed brain atrophy and microstructural damage of the NAWM on DTI analysis as demonstrated in Table 2. This suggests a potential direct effect of the ongoing atherosclerotic process on brain tissue. We hypothesize that the atherosclerotic tissue may directly promote pathological processes in the distal micro-circulation, leading to microangiopathic changes. Atherosclerotic tissue is a source of inflammatory activity that generates downstream micro-emboli containing fibrin, platelet clots, plaque debris, and inflammatory cytokines (42). These micro-emboli can accelerate pathological changes in the small penetrating arteries, leading to ischemic white matter changes and subsequent brain atrophy. Atherosclerosis-related luminal stenosis can induce hemodynamic changes, which in conjunction with small platelet aggregates or cholesterol micro-emboli shed from the diseased arterial wall, may play important roles in chronic cognitive decline, even in the absence of recurrent acute vascular occlusion.

Previous cross-sectional studies have shown an association between vascular calcifications and cognitive function (22). Our study supports these findings, demonstrating a strong association between the ICCS and cognitive function at enrollment, as well as at all follow-up time points up to 2 years following the index event, independent of age, gender, stroke severity, and known risk factors for PSCI. Furthermore, higher ICCS scores predicted and were associated with an increased likelihood of developing future PSCI and dementia.

An exploratory subgroup analysis was conducted on individuals who underwent a CTA, demonstrated that the degree of overall cranio-cervical vessel stenosis significantly correlated with the ICCS, as expected. However, it is noteworthy that even in individuals without arterial stenosis, the ICCS still retained its independent correlation with long-term PSCI. This finding may suggest that proximal atherosclerosis can influence the downstream vascular tree through mechanisms other than hemodynamic restriction secondary to luminal narrowing. The pathophysiology underlying the association between ICCS and cognition, whether through direct hemodynamic effects due to arterial stenosis or as a result of local plaque formation and stability, requires further investigation and additional studies are required to establish a causative relationship.

Our findings highlight the potential utility of the ICCS as an imaging marker for future brain dysfunction. In the field of cardiology, CAC has been established as a predictor of coronary stenosis and risk of myocardial infarction, and is routinely used for individual patient prognostication (14, 15). Similarly, the ICCS, which measures a similar phenomenon, may serve as a marker of intracranial vessel stenosis, brain tissue damage, and future PSCI. Previous cross-sectional analyses have shown the ICCS to be useful in post-stroke prognostication (22). Our study further suggests that the ICCS retains its predictive role for future PSCI, assessed up to 24 months after the index event. NCCT is a widely used and easily accessible imaging modality for stroke patients due to its speed, reliability, and cost-effectiveness. As such, the ICCS has potential usefulness as a readily available and rapidly obtainable marker of arterial calcifications. In our cohort it acted as a better predictor for PSCI compared to other well-known MRI imaging markers for PSCI. While the ICCS should not replace MRI as an important imaging modality, it may serve as a valuable bedside prognostic tool and help guide the need for aggressive risk factor management, similar to the CAC in the field of cardiology.

Our study has several limitations that should be taken into consideration. First, the ICCS is a crude measure that does not account for non-calcified “soft” atherosclerotic plaques, which are considered more unstable and may play a more important role in the pathophysiology of brain atherosclerosis. Second, although our data supports the association between ICCS and PSCI, we cannot establish a causative relationship. Additional limitations include the lack of follow-up imaging and the inclusion of only patients with mild clinical stroke manifestations.

Moreover, the majority of our study population consisted of Caucasians, which limits our ethnic diversity and the external validity of our findings. Larger studies with a more ethnically diverse population are needed to assess the generalizability and practical application of the ICCS in various ethnic groups. Lastly, even though the loss to follow-up was relatively modest, there was likely some selection bias regarding the long-term cognitive results. Multiple imputations were used to address this problem and provide an estimate of probable cognitive results in patients lost to follow-up. We used many predictor variables, demonstrating consistent results. Also, no differences were observed in baseline characteristics between participants included and not included in the current analysis. Thus, it seemed reasonable to assume that data were missing at random. Under the assumption of missing at random, we may obtain valid inferences by applying the multiple imputation technique. Analysis of the observed data set (including imputed values) revealed the same trend of results for cognitive scores.

The study has several strengths, including its prospective design, long-term detailed cognitive follow-up, and extensive and advanced MRI analyses for most participants.

In order to thoroughly assess the reliability of ICCS as an imaging marker for predicting cognitive prognosis and determine its practicality in clinical decision-making, along with establishing cut-off values for clinically significant ICCS in stroke survivors, it is imperative to conduct larger studies that encompass a more diverse ethnic population. Furthermore, future investigations should focus on exploring and validating a straightforward visual scoring system based on ICCS, which could potentially provide benefits to clinicians in a bedside setting.

## Conclusion

The ICCS is a simple and easily obtainable score based on semi-automatic analysis of NCCT. Our study found that in mild stroke survivors with intact cognition, ICCS was associated with brain atrophy, microstructural tissue damage, cerebral SVD, and higher rates of prospective PSCI. Furthermore, ICCS independently predicted cognitive deterioration 24 months after the stroke. The use of ICCS may help guide post-stroke prognostication and lead to individualized therapy by adjusting treatment targets in stroke survivors.

## Data availability statement

The original contributions presented in the study are included in the article/supplementary material, further inquiries can be directed to the corresponding author.

## Ethics statement

The studies involving human participants were reviewed and approved by the Tel-Aviv Sourasky medical center ethics committee. The patients/participants provided their written informed consent to participate in this study.

## Author contributions

ES: conceptualization, methodology, investigation, validation, supervision, writing original draft, review, and editing of final draft. US-G: methodology, investigation, validation, writing original draft, review, and editing of final draft. PB: methodology and software. IB and GS: investigation and software. BN: conceptualization, review, and editing of final draft. OR: methodology, review, and editing of final draft. JM: conceptualization, methodology, review, and editing of final draft. EBA: formal analysis, investigation, methodology, writing original draft, review, and editing of final draft. HH: conceptualization, formal analysis, supervision, writing original draft, review, and editing of final draft. All authors contributed to the article and approved the submitted version.

## Funding

The Tel Aviv brain acute stroke cohort (TABASCO) study (ClinicalTrials.gov #NCT01926691) was supported by grants RAG11482 from the American Federation for Aging Research (to EBA), 3-5062 from the Israeli Chief Scientist, Ministry of Health (to EBA), 2011344 From the U.S. – Israel Bi-national Science Foundation (to EBA), and AARG-16-442861 from the Alzheimer’s Association (to EBA).

## Conflict of interest

The authors declare that the research was conducted in the absence of any commercial or financial relationships that could be construed as a potential conflict of interest.

## Footnotes

^1^ http://surfer.nmr.mgh.harvard.edu/

^2^ http://www.fmrib.ox.ac.uk/fsl/

^3^ http://surfer.nmr.mgh.harvard.edu/
